# Influenza Vaccine Effectiveness in Preventing Severe Outcomes in Patients Hospitalized with Laboratory-Confirmed Influenza during the 2017–2018 Season. A Retrospective Cohort Study in Catalonia (Spain)

**DOI:** 10.3390/v13081465

**Published:** 2021-07-27

**Authors:** Lesly Acosta, Nuria Soldevila, Nuria Torner, Ana Martínez, Xavier Ayneto, Cristina Rius, Mireia Jané, Angela Domínguez, the Influenza Surveillance Network of Catalonia, PIDIRAC

**Affiliations:** 1 Department of Statistics and Operations Research, Polytechnic University of Catalonia/BARCELONATECH, 08028 Barcelona, Spain; lesly.acosta@upc.edu; 2 CIBER Epidemiología Salud Pública, CIBERESP, PREVICET Program, 28029 Madrid, Spain; nsoldevila@ub.edu (N.S.); a.martinez@gencat.cat (A.M.); crius@aspb.cat (C.R.); mireia.jane@gencat.cat (M.J.); angela.dominguez@ub.edu (A.D.); 3 Department of Medicine, University of Barcelona, 08036 Barcelona, Spain; 4 Public Health Agency of Catalonia, 08005 Barcelona, Spain; xavier.ayneto@gmail.com; 5 Public Health Agency of Barcelona, 08024 Barcelona, Spain

**Keywords:** influenza, intensive care unit, vaccine effectiveness, length of stay

## Abstract

Seasonal influenza is a common cause of hospital admission, especially in older people and those with comorbidities. The objective of this study was to determine influenza vaccine effectiveness (VE) in preventing intensive care admissions and shortening the length of stay (LOS) in hospitalized laboratory-confirmed influenza cases (HLCI) in Catalonia (Spain). A retrospective cohort study was carried out during the 2017–2018 season in HLCI aged ≥18 years from 14 public hospitals. Differences in means and proportions were assessed using a t-test or a chi-square test as necessary and the differences were quantified using standardized effect measures: Cohen’s d for quantitative and Cohen’s w for categorical variables. Adjusted influenza vaccine effectiveness in preventing severity was estimated by multivariate logistic regression where the adjusted VE = (1 − adjusted odds ratio) · 100%; adjustment was also made using the propensity score. We analyzed 1414 HLCI aged ≥18 years; 465 (33%) were vaccinated, of whom 437 (94%) were aged ≥60 years, 269 (57.8%) were male and 295 (63.4%) were positive for influenza type B. ICU admission was required in 214 (15.1%) cases. There were 141/1118 (12.6%) ICU admissions in patients aged ≥60 years and 73/296 (24.7%) in those aged <60 years (*p* < 0.001). The mean LOS and ICU LOS did not differ significantly between vaccinated and unvaccinated patients. There were 52/465 (11.2%) ICU admissions in vaccinated cases vs. 162/949 (17.1%) in unvaccinated cases. Patients admitted to the ICU had a longer hospital LOS (mean: 22.4 [SD 20.3] days) than those who were not (mean: 11.1 [SD 14.4] days); *p* < 0.001. Overall, vaccination was associated with a lower risk of ICU admission. Taking virus types A and B together, the estimated adjusted VE in preventing ICU admission was 31% (95% CI 1–52; *p* = 0.04). When stratified by viral type, the aVE was 40% for type A (95% CI -11–68; *p* = 0.09) and 25% for type B (95% CI -18–52; *p* = 0.21). Annual influenza vaccination may prevent ICU admission in cases of HLCI. A non-significantly shorter mean hospital stay was observed in vaccinated cases. Our results support the need to increase vaccination uptake and public perception of the benefits of influenza vaccination in groups at a higher risk of hospitalization and severe outcomes.

## 1. Introduction

Seasonal influenza is a common cause of hospital admission, especially in older people and those with comorbidities. Seasonal influenza epidemics cause an estimated 3–5 million severe illnesses and 290,000 to 650,000 deaths worldwide annually [1]. Severe outcomes are more frequently observed in the very young and older people, pregnant women, immunocompromised individuals, and patients of any age with chronic disease [2]. Despite the moderate overall protection, annual vaccination of these risk groups is the main preventive measure to avoid severity and hospitalization. Severity implies not only complications such as pneumonia, severe respiratory distress, multiorgan failure and death, but also intensive care unit (ICU) admission due to the complications of influenza infection. ICU admission is associated with adverse outcomes and excess health costs, especially longer hospital stays (LOS) [3].

Influenza is generally self-limiting and patients usually recover in about two weeks without medical care or antiviral drugs [1,2]. However, some groups are at higher risk for more severe disease, which may require hospitalization and/or ICU admission due to complications such as pneumonia or acute respiratory distress. Persons aged ≥65 years have the highest risk of increased morbidity including respiratory failure and mortality. It is estimated that >60% of all seasonal influenza-related hospitalizations and 90% of seasonal influenza-related deaths each year occur in older people [2,3,4,5,6,7]. Influenza vaccination can reduce influenza illness and, in turn, the primary healthcare workload, occupational and school absenteeism and influenza-related hospitalizations and deaths, especially in older people and those with underlying medical conditions. National recommendations on the annual influenza vaccination differs according to the country. In Catalonia (Spain), seasonal vaccination is recommended for persons in medical risk groups, pregnant women, people aged ≥60 years, and those with an occupational risk [4].

Since the 2009 A(H1N1)pdm09 influenza pandemic, several countries and regions have collected data on severe cases of influenza in order to assess the severity of influenza epidemics. The Public Health Agency’s Sub-directorate of Surveillance and Response to Public Heath Emergencies of Catalonia collects data on hospitalizations due to severe laboratory-confirmed influenza illness through a network of sentinel third-level hospitals covering approximately 62% of the population [5]. These data are used in turn for decision making on preventive strategies, diagnoses, and treatment. However, more detailed data are needed on hospitalizations for laboratory-confirmed influenza that are not classified upon admission as severe cases. Recognizing this need, the hospital sentinel surveillance network of the PIDIRAC (Daily Information Plan for Acute Respiratory Infections) expanded its registry to include all sentinel hospital emergency room admissions of laboratory-confirmed influenza cases [5].

In the influenza season studied, the predominant circulation was of the influenza B virus (63% of isolates) and the predominant lineage was Yamagata. The vaccine delivered to the population was the trivalent influenza vaccine, which contained a Victoria lineage. The A(H1N1)pdm09 strains were similar to previous seasons and concordant with the vaccine strain, while a proportion of H3N2 circulating strains differed from those contained in the trivalent vaccine [6].

The objective of this study was to describe the demographic, virological and clinical characteristics of hospitalized laboratory-confirmed influenza cases, including the LOS and ICU LOS, according to vaccination status. The primary research question was whether influenza vaccination prevented ICU admission of adults aged ≥18 years in the 2017–2018 influenza season in Catalonia. 

## 2. Materials and Methods

We carried out a retrospective cohort study of hospitalized laboratory-confirmed influenza cases aged ≥18 years, hospitalized for >24 h from 1 October 2017 to 22 May 2018, in the 14 tertiary hospitals comprising the Influenza Acute Respiratory Disease Surveillance Network of Catalonia (PIDIRAC). 

Two groups of samples were studied: Group 1 consisted of hospitalized severe laboratory-confirmed influenza cases (SHLCI) notified to the PIDIRAC. An SHLCI case was defined as a case of laboratory-confirmed influenza virus infection requiring hospitalization due to pneumonia, acute respiratory distress syndrome, septic shock, multiorgan failure, or other severe condition, or ICU admission, or who developed these criteria during hospitalization for other reasons. 

Group 2 consisted of laboratory-confirmed influenza cases recorded by the minimum hospital discharge data of the emergency room (CMBDH-ER) register according to the International Classification of Diseases (ICD-10) codes. Influenza cases were classified as: J09 Influenza due to certain identified influenza viruses—this includes the following types: A(H1N1)pdm09 and influenza of animal origin; J10 Influenza due to other identified influenza viruses—this includes any specified type not of animal origin and not listed as one of the types under the novel influenza A virus category, and, J11 Influenza due to unidentified influenza viruses—not documented as a specific type. Only cases admitted to the reporting hospital were included [7].

**Figure 1:**
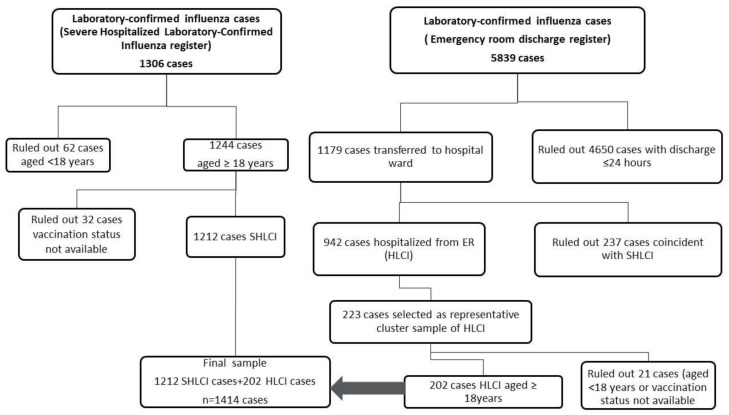
Patient selection flowchart of severe hospitalized laboratory-confirmed (SHLCI) and laboratory-confirmed influenza cases from the emergency room minimum hospital discharge dataset who remained hospitalized (HLCI). Catalonia, 2017–2018.

To assess coincident cases, files from the two databases were merged using the patient’s unique personal identifier. Of the non-coincident cases with ER discharge recovered after merging, a random sample stratified by hospital was selected to obtain a representative sample. The inclusion/selection of cases in the subset HCLI was made independently of the vaccination status (exposure) and the ICU admission (outcome) to avoid later bias in VE estimates. The difference in exposure due to age and hospital of recruitment was taken into account to minimize the bias. Data were then anonymized. The following clusters were predetermined: age, sex and epidemiological week of report, to preserve the representability of the population attended by each hospital (Figure 1).

Laboratory confirmation was made by polymerase chain reaction (PCR) and/or cultures of nasal aspirate or nasopharyngeal swab sampling, as described in previous reports [5,8,9].

We retrospectively obtained information on all cases from admission to discharge to determine the disease progression and outcome. The variables studied were collected from each reported case using a structured questionnaire for SHLCI cases [4]; for cases derived from ER discharge with no available questionnaire, the information was completed by consultation of medical records. Missing vaccination status on the questionnaire was ascertained by consultation of vaccine registers. For SHLCI and HLCI cases, we obtained the sex, age, pre-existing chronic diseases (chronic obstructive respiratory disease (COPD), obesity, diabetes, chronic kidney, cardiovascular and liver disease, immunodeficiency and other comorbidities, including hemoglobinopathies, severe neuromuscular diseases, impaired cognitive dysfunction), date of symptom onset and date of hospital and ICU admission and discharge, antiviral treatment received and whether treatment was <48 h or >48 h after symptom onset, seasonal influenza vaccination status and influenza virus type and subtype, when available.

### Statistical Analysis

Differences in means and proportions between vaccinated and nonvaccinated cases, with respect to independent variables (sociodemographic, virological, and clinical) and the LOS variables were assessed using the Student *t* test or chi-squared test as required. To quantify the differences, standardized effect size measures were used; Cohen’s d for quantitative and Cohen’s w for categorical variables (values of 0.10, 0.25, and 0.40 represent small, medium, and large effect sizes, respectively) [10]. Differences in means and proportions in cases admitted to the ICU or not and between SHLCI (group 1) and HLCI (group 2) cases were assessed.

The adjusted VE (aVE) associated with seasonal vaccination was estimated by a multivariate logistic regression model for ICU admission, adjusted by age, sex, ≥1 comorbidity, and timing of neuraminidase inhibitor treatment (NI). The aVE was calculated as (1-aOR) 100%. A logistic regression model adjusted by the propensity score, taking vaccination status as the outcome and based on the same adjusted covariates was constructed. The choice of potential confounders was guided by the statistics and expert knowledge of influenza disease. Overall estimates of aVE, in preventing ICU admission and estimates according to influenza virus type, age and sex were also calculated. The aVE for SLHCI and HLCI cases was calculated separately. The analysis was performed using the SPSS v.25 statistical package (IBM Corp. Released 2017. IBM SPSS Statistics for Windows, Version 25.0. Armonk, NY, USA: IBM Corp.), and R v3.6.2 statistical software (http://cran.r-project.org; accessed on 15 February 2020).

Ethical aspects: The information used in the study is part of routine monitoring in the surveillance of influenza as a public health activity and did not require informed consent. The final database was anonymized to preserve the confidentiality of patients.

## 3. Results

A total of 1529 (1306 SHLCI and 223 HLCI) cases were initially recorded: 115 cases were ruled out due to age <18 years (83 cases) or missing information on the vaccination status. Therefore, 1414 cases were included in the analysis: 465 (33%) were vaccinated cases of whom, 437 (94%) were aged ≥60 years, 269 (57.8%) were male and 295 (63.3%) had influenza type B. The influenza A subtype was available in 160 A cases (26.2%), of whom 90 (56.3%) were A (H1N1)pdm09 and 70 (43.7%) AH3N2. 

Of the cases hospitalized, 1127 (79.7%) had ≥1 underlying comorbidity, including 406/465 (87.3%) in vaccinated cases and 721/949 (76%) in unvaccinated cases (*p* < 0.001). The most frequent comorbidity was cardiovascular disease in 614 cases (43.4%), with a vaccine coverage of 38.6% (237/614). Of vaccinated cases, 40.9% (190/465) had COPD, compared with 28.6% (271/949) in unvaccinated cases (*p* < 0.001). 

In the 63 women of childbearing age (18–50 years) included, there were 8 pregnancies (12.7%) with 2/6 (33.3%) in vaccinated cases and 6/57 (10.5%) in unvaccinated cases (*p* = 0.11). At discharge, there was no significant difference in the mean LOS according to the vaccination status (12.1 days (SD 14.1) in vaccinated patients and 13.1 days (SD 16.8) in unvaccinated cases (*p* = 0.228). The mean ICU LOS was 9.5 days (SD 9.3) in vaccinated cases and 9.3 days (SD 10.8) in unvaccinated cases (*p* = 0.917). Cohen’s effect measures showed almost all variables considered had relatively small effects; with age, age group and ≥1 comorbidity having the largest effects (0.62, 0.23 and 0.13, respectively) (Table 1). 

The mean age of cases was 71.5 years (SD 15.2), which was higher in SHLCI cases than in HLCI cases (71.1 years (SD15.3) vs. 68.5 years (SD 16); *p* = 0.008, respectively). There were fewer SHLCI with ≥1 comorbidity (77.9% vs. 91.4%; *p* < 0.001) and fewer people aged >60 years (78.2% vs. 85%; *p* = 0.040), while seasonal vaccination coverage (35.9% vs. 13.4%; *p* < 0.001) and NI treatment was higher in the SHLCI group (93.4% vs. 81.8%; *p* < 0.001). The difference in effect size was small, <0.20, for these results (Table 2).

ICU admission was required by 214 cases (15.1%), with a mean age of 64 years (SD 13.6) compared with 72.8 years (SD 15.1) for non- ICU cases (*p* < 0.001). Underlying chronic liver disease was more frequent in ICU patients (24/88, 27.3%). Age >60 years was less frequent in ICU patients: ICU admissions in patients aged ≥60 years were (141/1118, 12.6%) compared with 73/296 (24.7%) in patients aged <60 years. Males were more frequently admitted to the ICU (135/804, 16.8%). Fewer vaccinated cases (52/465, 11.2%) than unvaccinated cases (162/949, 17.1%) required ICU admission. In the 63 women of childbearing age (18–50 years) included, there were 8 pregnancies (12.7%) with 2/14 (14.3%) admitted to the ICU vs. 6/49 (12.2%) who were not (*p* = 0.83). Patients admitted to the ICU had a longer LOS than those who were not (22.4 (SD 20.3) days vs. 11.1 (SD 14.4) days; *p* < 0.001) (Table 3).

The estimated VE obtained by multivariate logistic regression, adjusting by variables of age, sex, presence of comorbidities and timing of NI treatment, is shown in Table 4. A propensity score model showed similar results (data not shown). Vaccination was associated with a lower risk of ICU admission, with an aVE of 31% (95% CI 1–52; *p* = 0.04). When stratified by viral type, the aVE was 40% for type A (95% CI -11–68; *p* = 0.09) and 25% for type B (95% CI -18–52; *p* = 0.21). Males aged ≥60 years admitted to the ICU were lower in vaccinated (31; 12.2%) than in unvaccinated cases (60; 15.9%) with aVE of 32% (95% CI -10–58). Other aVE according to age and sex are shown in Table 4.

There were differences in the effectiveness of seasonal influenza vaccination in the SHLCI and HLCI groups. Crude VEs were 47% (95% CI 25–62) for the SHLCI group and 31% (95% CI -578–99.5) for the HLCI group. When sample size allowed, adjusted aVE were calculated. The overall aVE was 31% (95% CI 1–52; 0.04) and was 40% (95% CI 13–58; *p* = 0.007) for the SHLCI group. The aVE was not computable for HLCI cases due to the small sample size (Table 5). 

## 4. Discussion

The 2017–2018 influenza season was a B lineage-mismatched season, with predominantly influenza B/Yamagata viruses circulating and a B/Victoria lineage virus included in the trivalent vaccine. Our results, as in other European countries [11], suggest that vaccination offered only slight protection against severe outcomes because of the nature of the circulating viruses, the high proportion of hospitalizations in older people (79.1%) and because older age is associated with lower ICU admission [12]. The inverse relationship between age and ICU admission has been shown by other studies, which found a higher rate of ICU admission in SHLCI cases aged 15–64 years [13,14,15,16]. Although vaccine effectiveness to prevent ICU admission can be impaired by co-infection with other respiratory agents (bacterial, viral or fungal) because of increased severity [17,18], we found that vaccinated patients had a lower risk of ICU admission risk than unvaccinated patients. Overall, vaccination reduced ICU admission with an aVE of 31% (*p* = 0.04), similar to the results reported by Arriola et al. during the 2013–2014 season (37%) [19].

In Catalonia, influenza vaccine is recommended in persons aged ≥60 years, risk groups of any age with comorbidities (COPD, cardiovascular disease, diabetes, immunodeficiency, obesity, and other chronic conditions), pregnant women, and health care workers [4]. However, vaccination coverage in among risk groups is below the Venice Network of the European Center for Disease Control (ECDC) recommended target of a coverage of 75% [20]. The reported vaccine coverage for this season in Catalonia was 55.7% in persons aged ≥60 years, similar to the 55% coverage reported in Spain and above the median of 47.1% reported in the European Union, but still below the ECDC target [20]. In persons aged <60 years with chronic conditions, the estimated coverage in Catalonia was 20%, compared with a median coverage of 44.9% in some European countries [4,21]. Our results show that 79.7% (1127/1414) of adults hospitalized with confirmed influenza had ≥1 comorbidity but only 36% were vaccinated against influenza. A similar coverage was found for patients with comorbidities, ranging from 29.6% in immunocompromised patients to 41.2% in COPD patients, far from the recommended 75% target. 

We found that 15.1% of cases required ICU admission, in agreement with other countries such as Ireland (16%) and the 15% reported by Lina et al. in a study of 14 countries in the 2017–2018 season [22,23]. 

The most common underlying medical condition was chronic liver disease, while other studies found COPD was one of the major comorbidities in hospitalized influenza patients requiring ICU admission [24]. Influenza can cause severe illness and ICU admission even in individuals without known comorbidities, as shown by our results (17.4%) and those of Lina et al. (15.3%) [23]. 

We found no differences with respect to reducing LOS and ICU LOS, in contrast to the findings by Arriola et al. in two seasons, where vaccination shortened both hospital and ICU LOS. However, the shorter LOS in patients not admitted to the ICU showed an indirect effect of vaccination in preventing ICU admission [19,25]. ICU LOS is an uncertain variable, subject to individual hospital policies. A study by Garland and Connors found there was an optimal time for patients to leave the ICU, with an increasing risk of subsequent death if patients leave too early or too late [26]. Clinical judgment may not be reliable in determining the optimal time window and intensivists use subjective clinical judgments to guide them in determining when patients should be admitted and discharged from the ICU [27].

In pregnant women hospitalized for influenza, although the small sample size did not permit significant results, some vaccination protection against ICU admission was observed. In accordance with Mazagatos et al., the results suggest that pregnant women could benefit from seasonal influenza vaccination [28]. There is a need for larger studies in this group, including various seasons and countries, in the line with the PREVENT protocol described by Naleway et al., who assessed the inactivated influenza vaccine effectiveness in preventing severe influenza during pregnancy [29].

One limitation of the study may be the disparity between the two groups, although the main outcome measure, influenza VE in preventing ICU admission, did not differ widely between the SHLCI and HCLI cases (VE 47% vs. 31%, respectively). Despite some significant differences between the two groups, the effect size was relatively low, reflecting small differences and the direction of the estimated effects was the same for both groups. Another possible limitation is that calendar time was not included in the VE analysis, but this does not alter the VE estimates because all cases included were laboratory confirmed, minimizing possible interactions with other respiratory viruses during the epidemic season, and because the temperate climate of Catalonia allows for vaccination prior to the onset of epidemic activity [30]. Our findings represent a single season and should be considered with caution when generalizing the results. 

In conclusion, our results show that influenza vaccination reduced the need for ICU admission and the length of hospitalization in cases of laboratory-confirmed influenza virus infections detected during the 2017–2018 influenza season. These findings support the need to increase vaccination uptake in elderly patients, pregnant women, and persons with at least one comorbidity. Further efforts are needed to increase public perceptions of the benefits of influenza immunization in groups at higher risk of hospitalization and severe outcomes.

## Figures and Tables

**Table 1:** Hospitalized cases of laboratory-confirmed influenza according to influenza vaccination status. Catalonia, 2017–2018.

Patient Parameters	Total Number of Hospitalized Influenza Cases *n* = 1414	Vaccinated Cases *n* = 465 (32.9%)	Unvaccinated Cases *n* = 949 (67.1%)	*p* Value ^a^	d-Cohen/ w-Cohen
**Age mean (SD) ^b^**	71.5 (15.2)	77.6 (11.3)	68.5 (16.0)	<0.001	0.62
**Age group**					
≥60 years	1118 (79.1%)	437 (94.0%)	681 (71.8%)	0.000	0.26
18–59 years	296 (20.9%)	28 (6.0%)	268 (28.2%)		
**Sex**					
Male	804 (56.9%)	269 (57.8%)	535 (56.4%)	0.600	0.01
Female	610 (43.1%)	196 (42.2%)	414 (43.6%)		
**Comorbidities (≥1)**					
Yes	1127 (79.7%)	406 (87.3%)	721 (76.0%)	<0.001	0.13
No	287 (20.3%)	59 (12.7%)	228 (24.0%)		
**COPD ^c^**					
Yes	461 (32.6%)	190 (40.9%)	271 (28.6%)	<0.001	0.12
No	953 (67.4%)	275 (59.1%)	678 (71.4%)		
**Obesity (BMI > 40) ^d^**					
Yes	111 (7.85%)	37 (8.0%)	74 (7.8%)	0.909	0.003
No	1303 (92.1%)	428 (92.0%)	875 (92.2%)		
**Diabetes**					
Yes	385 (27.2%)	141 (30.3%)	244 (25.7%)	0.069	0.05
No	1029 (72.8%)	324 (69.7%)	705 (74.3%)		
**Chronic renal disease**					
Yes	275 (19.4%)	106 (22.8%)	169 (17.8%)	0.028	0.06
No	1139 (80.6%)	359 (77.2%)	780 (82.2%)		
**Immunodeficiency**					
Yes	230 (16.3%)	68 (14.6%)	162 (17.1%)	0.242	0.03
No	1184 (83.7%)	397 (85.4%)	787 (82.9%)		
**Cardiovascular disease**					
Yes	614 (43.4%)	237 (51.0%)	377 (39.7%)	<0.001	0.10
No	800 (56.6%)	228 (49.0%)	572 (60.3%)		
**Chronic liver disease**					
Yes	88 (6.22%)	28 (6.0%)	60 (6.3%)	0.836	0.006
No	1326 (93.8%)	437 (94.0%)	889 (93.7%)		
**Other comorbidities ^e^**					
Yes	165 (11.7%)	66 (14.2%)	99 (10.4%)	0.041	0.06
No	1249 (88.3%)	399 (85.8%)	850 (89.6%)		
**NI ^f^ treatment**					
Yes	1299 (91.9%)	437 (94.0%)	862 (90.9%)	0.045	0.05
**NI ^f^ treatment (timing)**					
≤48 h from onset of symptoms	511 (36.9%)	169 (36.3%)	342 (36.1%)	0.075	0.05
>48 h from onset of symptoms	759 (54.8%)	257 (55.3%)	502 (53.0%)	0.046	
No	114 (8.1%)	28 (6.0%)	86 (9.1%)		
**Type of influenza virus**					
B	859 (60.9%)	295 (63.4%)	564 (59.7%)	0.174	0.04
A	551 (39.1%)	170 (36.6%)	381 (40.3%)		
**Outcome variables:**					
**ICU admission**					
Yes	214 (15.1%)	52 (11.2%)	162 (17.1%)	0.005	0.08
No	1200 (84.9%)	413 (88.8%)	787 (82.9%)		
**LOS ^g^**					
Mean days (SD)	12.8 (15.9)	12.1 (14.1)	13.1 (16.8)	0.228	0.06
**ICU LOS ^h^**					
Mean days (SD)	9.35 (10.4)	9.49 (9.30)	9.31 (10.8)	0.917	0.02

^a^ Student *t* or chi-square/Fisher’s test used as appropriate; ^b^ SD: Standard deviation; ^c^ COPD: Chronic obstructive pulmonary disease; ^d^ BMI: Body mass index; ^e^ Other comorbidities include hemoglobinopathies and cognitive impairment; ^f^ NI: Neuraminidase inhibitor; ^g^ LOS: Length of hospital stay; ^h^ ICU LOS: Length of intensive care unit stay; Column percentages reported.

**Table 2:** Main characteristics of hospitalized influenza by source: Severe hospitalized laboratory-confirmed. Influenza (SHLCI) and hospitalized laboratory-confirmed influenza from emergency room discharge data (HLCI). Catalonia, 2017–2018.

Patient Parameters	SHLCI ^a^ *n* = 1227	HLCI ^b^ *n* = 187	*p* Value	d-Cohen/ w-Cohen
**Age, mean (SD) ^c^**	71.1 (15.3)	68.5 (16.0)	0.008 *	0.17
**Age group**				
≥60 years	959 (78.2%)	159 (85.0%)	0.040	0.06
18–59 years	268 (21.8%)	28 (15.0%)		
**Sex**				
Male	709 (57.8%)	95 (50.8%)	0.086	0.05
Female	518 (42.2%)	92 (49.2%)		
**Comorbidities (≥1)**				
Yes	956 (77.9%)	171 (91.4%)	<0.001	0.11
No	271 (22.1%)	16 (8.56%)		
**Influenza vaccination**				
Yes	440 (35.9%)	25 (13.4%)	<0.001	0.16
No	787 (64.1%)	162 (86.6%)		
**NI ^d^ treatment**				
Yes	1146 (93.4%)	153 (81.8%)	<0.001	0.14
No	81 (6.60%)	34 (18.2%)		
**NI ^d^ treatment (timing)**				
≤48 h from symptom onset	452 (37.7%)	59 (31.7%)	<0.001	0.14
>48 h from symptom onset	665 (55.5%)	94 (50.5%)		
No	81 (6.8%)	33 (17.7%)		

^a^ SHLCI: Severe hospitalized laboratory-confirmed influenza; ^b^ HLCI: Hospitalized laboratory-confirmed influenza from emergency room discharge data; ^c^ SD: Standard deviation; ^d^ NI: Neuraminidase inhibitor; * Student *t* test. Column percentages reported.

**Table 3:** Hospitalized influenza cases according to intensive care unit admission. Catalonia, 2017–2018.

Patient Parameters	ICU Admission *n* = 214 (15.1%)	No ICU Admission *n* = 1200 (84.9%)	*p* Value	d-Cohen/ w-Cohen
**Influenza vaccination**				
Yes	52 (11.2%)	413 (88.8%)	0.003	0.08
No	162 (17.1%)	787 (82.9%)		
**Age**				
Years, mean (SD) ^b^	64.0 (13.6)	72.8 (15.1)	<0.001 *	0.59
**Age group**				
≥60 years	141 (12.6%)	977 (87.4%)	<0.001	0.14
18–59 years	73 (24.7%)	223 (75.3%)		
**Sex**				
Male	135 (16.8%)	669 (83.2%)	0.046	0.05
Female	79 (13.0%)	531 (87.0%)		
**Comorbidities**				
Yes	164 (14.6%)	963 (85.4%)	0.230	0.03
No	50 (17.4%)	237 (82.6%)		
**COPD ^c^**				
Yes	74 (16.1%)	387 (83.9%)	0.502	0.02
No	140 (14.7%)	813 (85.3%)		
**Obesity (BMI > 30) ^d^**				
Yes	23 (20.7%)	88 (79.3%)	0.098	0.05
No	191 (14.7%)	1112 (85.3%)		
**Diabetes**				
Yes	60 (15.6%)	325 (84.4%)	0.767	0.01
No	154 (15.0%)	875 (85.0%)		
**Chronic renal disease**				
Yes	31 (11.3%)	244 (88.7%)	0.043	0.05
No	183 (16.1%)	956 (83.9%)		
**Immunodeficiency**				
Yes	33 (14.3%)	197 (85.7%)	0.728	0.01
No	181 (15.3%)	1003 (84.7%)		
**Cardiovascular disease**				
Yes	79 (12.9%)	535 (87.1%)	0.037	0.05
No	135 (16.9%)	665 (83.1%)		
**Chronic liver disease**				
Yes	24 (27.3%)	64 (72.7%)	0.002	0.09
No	190 (14.3%)	1136 (85.7%)		
**Other comorbidities ^e^**				
Yes	27 (16.4%)	138 (83.6%)	0.630	0.01
No	187 (15.0%)	1062 (85.0%)		
**NI ^f^ treatment**				
Yes	199 (15.3%)	1100 (84.7%)	0.552	0.02
No	15 (13.2%)	99 (86.8%)		
**NI ^f^ treatment (timing)**				
≤48 h from symptom onset	62 (12.1%)	449 (87.9%)	0.750	0.06
>48 h from symptom onset	128 (16.9%)	631 (83.1%)	0.323	
No	15 (13.2%)	99 (86.8%)		
**Influenza virus type**				
A	92 (16.7%)	459 (83.3%)		
B	122 (14.2%)	737 (85.8%)	0.205	0.04
**LOS ^g^**				
Mean days (SD) ^b^	22.4 (20.3)	11.1 (14.4)	<0.001 *	0.73

^a^ ICU: Intensive care unit; ^b^ SD: Standard deviation; ^c^ COPD = Chronic obstructive pulmonary disease; ^d^ BMI: Body mass index; ^e^ Other comorbidities: include hemoglobinopathy, severe neurological disorder and cognitive impairment; ^f^ NI: Neuraminidase inhibitor; ^g^ LOS: Length of hospital stay; * Student *t* test; Row percentages reported.

**Table 4:** Vaccine effectiveness in preventing intensive care unit admission of hospitalized cases of laboratory-confirmed influenza according to virus type and age and gender group. Catalonia, 2017–2018.

All Patients	ICU Admission	No ICU Admission	aVE ^a^	*p* Value
***n* = 1414**	***n* = 214 (15.1%)**	***n* = 1200 (84.9%)**	**(95%CI) ^b^**	
**Influenza vaccination** **Vaccinated (465; 32.9%)**	52 (11.2%)	413 (88.8%)	31% (1; 52)	0.04
Unvaccinated (949; 67.1%)	162 (17.1%)	787 (82.9%)	Ref.	
**Influenza B** ***n* = 859 (60.7%)**				
Vaccinated (295; 34.3%)	33 (11.2%)	262 (88.8%)	25% (−18; 52)	0.21
Unvaccinated (564; 65.7%)	89 (15.8%)	475 (84.2%)	Ref.	
**Influenza A** ***n* = 551 (38.9%)**				
Vaccinated (170; 30.9%)	19 (11.2%)	151 (88.8%)	40% (−11; 68)	0.09
Unvaccinated (381; 69.1%)	73 (19.2%)	308 (80.8%)	Ref.	
**Female, age <60 years** ***n* = 125 (8.8%)**				
Vaccinated (14; 11.2%)	1 (7.1%)	13 (92.9%)	72% (−135; 97)	0.24
Unvaccinated (111; 88.8%)	28 (25.2%)	83 (74.8%)	Ref.	
**Female, age ≥60 years** ***n* = 485 (34.3%)**				
Vaccinated (182; 37.5%)	17 (9.3%)	165 (90.7%)	23% (−48; 60)	0.43
Unvaccinated (303; 62.5%)	33 (10.9%)	270 (89.1%)	Ref.	
**Male, age <60 years** ***n* = 171 (24.2%)**				
Vaccinated (14; 8.2%)	3 (21.4%)	11 (78.6%)	29% (−74; 82)	0.62
Unvaccinated (157; 91.8%)	41(26.1%)	116 (73.9%)	Ref.	
**Male, age ≥60 years** ***n* = 633 (44.7%)**				
Vaccinated (255; 40.3%)	31 (12.2%)	224 (87.8%)	32% (−10; 58)	0.12
Unvaccinated (378; 59.7%)	60(15.9%)	318 (84.1%)	Ref.	

^a^ aVE: estimated VE adjusted by age, sex, ≥1 comorbidity and NI treatment (if yes, administered < or >48 h after symptom onset); ^b^ 95%CI: Wald confidence interval. Row percentages reported.

**Table 5:** Vaccine effectiveness (overall and according to data sources) in preventing intensive care unit admission in hospitalized laboratory-confirmed influenza cases. Catalonia, 2017–2018.

	ICU Admission	No ICU Admission	VE *	aVE **	*p* Value
**All Patients *n* = 1414**	***n* = 214 (15.1%)**	***n* = 1200 (84.9%)**	**(95% CI)**	**(95% CI)**	
**Influenza vaccination**					
Vaccinated (465; 32.9%)	52 (11.2%)	413 (88.8%)	39% (15; 56)	31% (1; 52)	0.040
Unvaccinated (949; 67.1%)	162 (17.1%)	787 (82.9%)		Ref.	
**SHLCI ^a^ *n* = 1227**	**210 (17.1%)**	**1017 (82.9%)**			
Vaccinated (440: 35.9%)	52 (11.8%)	388 (88.2%)	47% (25; 62)	40% (13; 58)	0.007
Unvaccinated (787; 64.1%)	158 (20.1%)	629 (79.9%)			
**HLCI ^b^ *n* = 187**	**4 (2.1%)**	**183 (97.9%)**			
Vaccinated (25; 13.3%)	0 (0.0%)	25 (100%)	31% (−578; 99.5) ***	Not computable	----
Unvaccinated (162; 86.7%)	4 (2.5%)	158 (97.5%)			

* Crude VE; ** VE adjusted by age, sex, ≥1 comorbidity and NI treatment. NI: Neuraminidase inhibitor ^a^ SHLCI: Severe hospitalized laboratory-confirmed influenza; ^b^ HLCI: Hospitalized laboratory-confirmed influenza; *** Firth’s penalized logistic regression used to compute crude VE. Row percentages reported.

## Data Availability

The data presented in this study are available on request from the corresponding author.
